# Microstructural Anisotropy of Magnetocaloric Gadolinium Cylinders: Effect on the Mechanical Properties of the Material

**DOI:** 10.3390/ma9050382

**Published:** 2016-05-17

**Authors:** Darja Steiner Petrovič, Roman Šturm, Iztok Naglič, Boštjan Markoli, Tomaž Pepelnjak

**Affiliations:** 1 Institute of Metals and Technology, Ljubljana 1000, Slovenia; darja.steiner@imt.si; 2 Faculty of Mechanical Engineering, University of Ljubljana, Ljubljana 1000, Slovenia; tomaz.pepelnjak@fs.uni-lj.si; 3 Faculty of Natural Sciences and Engineering, University of Ljubljana, Ljubljana 1000, Slovenia; iztok.naglic@omm.ntf.uni-lj.si (I.N.); bostjan.markoli@omm.ntf.uni-lj.si (B.M.)

**Keywords:** magnetocaloric, Gadolinium, inclusions, selective oxidation, mechanical properties

## Abstract

The development of advanced materials and technologies based on magnetocaloric Gd and its compounds requires an understanding of the dependency of mechanical properties on their underlying microstructure. Therefore, the aim of the study was to characterize microstructural inhomogeneities in the gadolinium that can be used in magnetocaloric refrigeration systems. Microstructures of magnetocaloric gadolinium cylinders were investigated by light microscopy and FE-SEM (Field Emission Scanning Electron Microscopy), EDS (Energy-dispersive X-ray Spectroscopy), and BSE (Back-scattered Electrons) in both the extrusion and the extrusion-transversal directions. XRD (X-ray Diffraction) analyses were performed to reveal the presence of calcium- and fluorine-based compounds. Metallographic characterization showed an oxidized and inhomogeneous microstructure of the cross-sections. The edges and the outer parts of the cylinders were oxidized more intensively on the surfaces directly exposed to the processing tools. Moreover, a significant morphological anisotropy of the non-metallic inclusions was observed. CaF inclusions act as active nucleation sites for internal oxidation. The non-metallic, Ca- and F-containing inclusions can be classified as complex calciumoxyfluorides. The solubility of Er and Yb in the CaF was negligible compared to the Gd matrix and/or the oxide phase. Lower mechanical properties of the material are a consequence of the lower structural integrity due to selective oxidation of surfaces and interfaces.

## 1. Introduction

The design of an efficient magnetic cooling system is a highly interdisciplinary challenge. A study of the literature shows that gadolinium (Gd) metal and its alloys are widely accepted as the reference material in magnetocaloric refrigeration systems [1,2,3,4,5,6,7,8,9]. Among alternative cooling and near-room temperature refrigeration technologies, magnetic refrigeration has the highest level of current research activity, and is considered to hold some, but not imminent, promise for implementation as better magnetocaloric materials and other technical breakthroughs are needed to realize this promise [10,11,12,13,14,15].

Generally, active magnetic refrigerators can be divided into two groups: ordered structures (parallel plate) and packed-bed structures (spheres, powder and cylinders) [8,9]. For micro-refrigeration devices, Gd thin films are of particular interest [16]. Gadolinium metal foil also appears as a candidate for modern hybrid undulators [17].

However, one of the most important factors when choosing Gd as a magnetocaloric material is its purity [18,19]. There have been few detailed studies highlighting the metallic impurities in Gd. These studies have predominantly focused on the interstitial impurities, such as O, N, and C. The various impurities present in Gd metal can be classified as follows: (i) rare-earth metallic impurities; (ii) alkali and alkali-earth metallic impurities; and (iii) others, e.g., Ti impurities [18]. Recently, an effective approach to prepare high purity Gd via hydrogen in-situ refining method for removing O and N was proposed in detail by Li *et al.* [19].

The (structural) inhomogeneities in Gd alloy systems may influence their magnetic properties [20,21,22]. The microstructure, *i.e.*, the fraction of grain boundaries, was found to critically influence the physico-chemical properties of bulk Gd metal [23,24]. With a decrease in the grain size from the micrometer to the nanometer range, (i) the Curie temperature decreased and exhibited a broader shape in DSC (Differential Scanning Calorimetry) heating curves [23] (ii) the room-temperature electrical resistivity was found to increase, while the low-temperature resistivity increased remarkably [24]. The oxidized surface layer of a ternary Gd alloy was not found to significantly affect the bulk magnetocaloric performance of the material [12].

In addition to gadolinium and other rare-earth alloy systems, calcium fluorides are also compounds with a relevance to materials science. CaF_2_ is a non-toxic, highly-transparent material, very attractive for improved laser materials as a matrix suitable for doping with rare earths [25,26]. Oxyfluoride glass ceramics have been given much attention due to their combined advantages of being oxide glasses and fluoride glasses, especially when doped with rare-earth ions [27,28].

The properties of magnetocaloric materials are sensitive to changes in structure in all scales [2]. Among most important criteria to identify the best material for magnetic refrigeration are: large magnetocaloric effect at room temperature and at moderate magnetic field, high chemical stability and good corrosion resistance of the magnetocaloric material, as well as limited number of elements in the compound in order to facilitate the control of reproducibility [14]. Magnetocaloric characteristics, *i.e.*, maximum temperature span and the heat-transfer performance of the gadolinium cylinders, have been previously investigated and published elsewhere [8]. Results reveal that the geometry of the magnetocaloric material has a crucial impact on the performance of the magnetic refrigerator. Samples with higher degree of deformation show reduction in magnetization in comparison with the polycrystalline Gd [29].

Therefore, the main goal of the present work is the additional characterization of the material, the metallographic determination of (anisotropic) microstructural inhomogeneities and their influence on the mechanical properties of commercial Gd cylinders that can be used as packed-bed AMRs [8]. Furthermore, the effect of microstructure on the mechanical properties of the material is discussed.

## 2. Experimental

**Figure 1:**
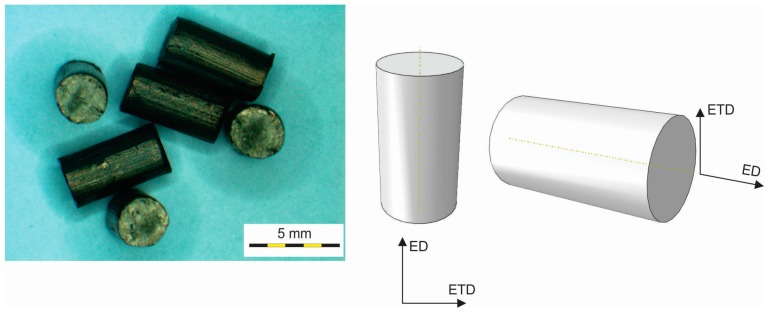
Photograph and schematic illustrations of Gd cylinders.

Metallographic analyses and mechanical testing under compressive loading of the commercial gadolinium cylinders presented in Figure 1 were performed to characterize the microstructure and mechanical properties of the material. The extrusion longitudinal direction (ED) and the extrusion transversal direction (ETD) are also shown in Figure 1. The geometrical properties of the gadolinium cylinders were: (a) Diameter: *d* = 2.88 to 2.94 mm; (b) Length: *L* = 5.31 to 5.53 mm.

### 2.1. Metallographic Analyses

For the metallographic analyses the specimens were ground and polished in accordance with standard metallographic techniques. The metallographic analyses were performed using light microscopy and scanning electron microscopy. For the light microscopy we used a Nikon Microphot FXA microscope (Nikon Instruments Europe B.V., Vienna, Austria). The field-emission scanning electron microscope was a JEOL JSM 6500f (JEOL Ltd., Tokyo, Japan) equipped with an energy-dispersive spectrometer. The FE-SEM, EDS, BSE analyses were performed at a 15-kV accelerating voltage. The representative specimens of commercial specimens of gadolinium cylindrical cylinders were analyzed in both the extrusion and extrusion transversal directions (Figure 1).

### 2.2. XRD Phase Analysis

XRD analysis has been performed on the extruded specimen along the direction of the extrusion, using PANalytical X´Pert PRO (PANalytical B.V., Almelo, The Netherlands) equipped with HighScore Plus software (PANalytical B.V., Almelo, The Netherlands) for refinement.

### 2.3. Mechanical Testing

The specimens used were as-received commercial Gd cylinders. The compressive properties at room temperature were analyzed using five specimens for each testing procedure. The mechanical testing using compression loading was performed in the extrusion direction (ED) and the extrusion transversal direction (ETD) of the cylinders (Figure 1). A Messphysik Beta 50-4/6×14, 50 kN universal testing device (Messphysik, Fürstenfeld, Austria) was used. The cropped specimens made of as-extruded substrate having a diameter of 2.91 ± 0.03 mm were cut on a lathe to obtain a regular cylindrical shape. The elastic properties of the material were obtained by upsetting in the extrusion direction as well as by compression in the transverse direction, while the flow curve of the material was determined for the extrusion direction only. In order to minimize the influence of friction the specimens were placed between a polytetrafluoroethylene (PTFE) foil during the upsetting tests. The friction coefficient of the PTFE foil was determined experimentally by Golchin *et al.* [30].

In the determination of the true-stress/true-strain curve an average stress value was calculated using Equation (1):

(1) $$\sigma_{f}=\frac{F}{A}$$

where *F* represents the measured load and *A* the current cross-section of the specimen.

The true compressive strain *ε_e_* (Equation (2)) was calculated as:

(2) $$\varepsilon_{e}=\ln\left(\frac{h_{0}}{h}\right)$$

When the loading was performed in the extrusion direction (ED) the length (*L*) of the cylindrical Gd cylinders was taken as the initial specimen height (*h*_0_), whereas the diameter (*d*) was taken as the initial height when the loading was performed in the extrusion transversal direction (ETD). The Young’s modulus was extracted for the extrusion direction from the elastic part of the stress-strain curve using a linear-regression tool. Since the specimens were too small to cut them out of the cylindrical specimen, also in the transverse direction, as shown in Figure 1, the transverse upsetting direction was used for the determination of the extrusion transversal direction Young’s modulus. Further, the yield point *σ_f_*_0_ was acquired according to the rule of 0.2% of plastic deformation determined regarding to the linear approximation of the elastic part of the stress-strain diagram. The stress from Equation (1) was calculated from the integral value of the cross-section on which the load was acting.

## 3. Results

### 3.1. Metallography

The identification and the characterization of the non-metallic inclusions were performed on selected commercial specimens of gadolinium cylindrical cylinders. The representative non-metallic inclusions were analyzed in both the extrusion and extrusion transversal directions (Figure 1).

**Figure 2:**
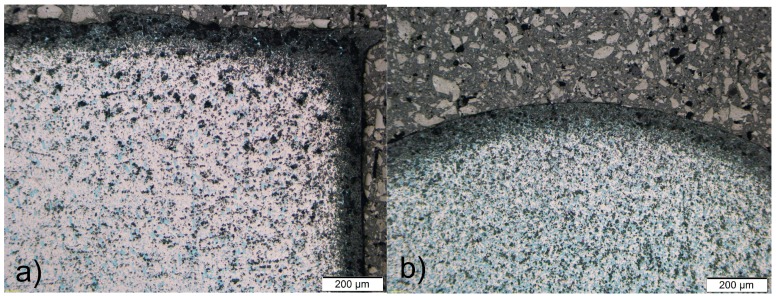
Non-homogeneous microstructure of the Gd cylinders (LM, polished). (**a**) Extrusion direction; and (**b**) extrusion transversal direction.

Figure 2 shows the non-homogeneous microstructure across the cross-sections of the gadolinium cylinders in both the extrusion and the extrusion transversal directions.

**Figure 3:**
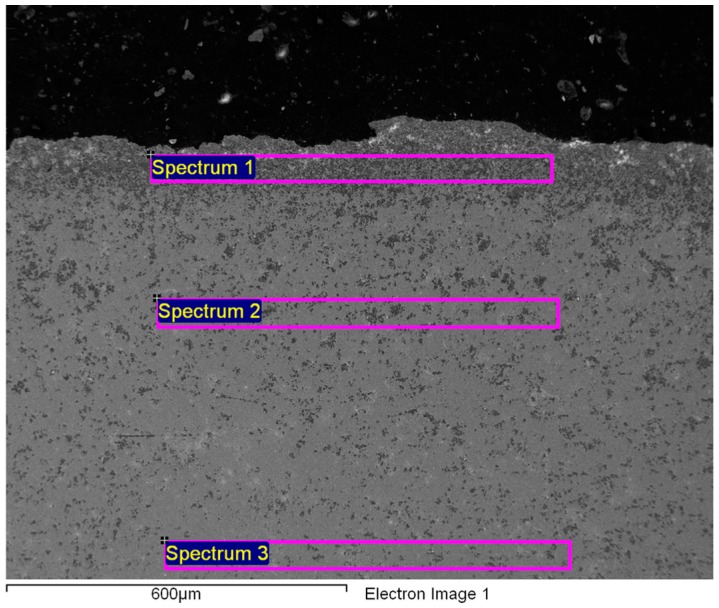
Secondary Electron (SE) image of the matrix and the edges of a Gd cylinder (extrusion direction, polished).

**Figure 4:**
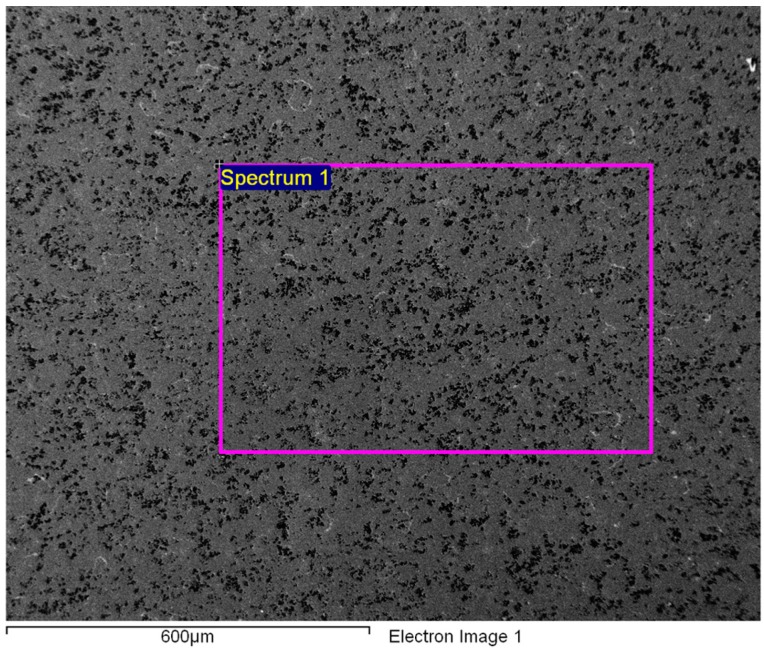
SE image of the matrix in the centre of a Gd cylinder (extrusion transversal direction, polished).

It is clear that the microstructure is very inhomogeneous. The edges and the outer parts of the specimens are oxidized more intensively on the surfaces directly exposed to the processing tools during the extrusion and the cutting (Figure 2, Figure 3 and Figure 4).

The chemical compositions of the rather inhomogeneous Gd cylinders may differ across the cross-sections as a result of surface oxidation. This is particularly evident from the EDS results given in Table 1 and Table 2. An increased content of oxygen in the centre of the Gd cylinder taken from the extrusion transversal direction is a consequence of the surface oxidation and the partial removal of the oxide layer by metallographic grinding and polishing (Figure 4, Table 2).

It is clear from Figure 2 and Figure 3, as well as from the EDS listed in Table 1, that especially the edges of the as-extruded specimen are oxidized to a greater extent. The FE-SEM/EDS results also reveal the very non-homogeneous chemical composition of the as-extruded Gd specimens across their cross-sections.

**Figure 5:**
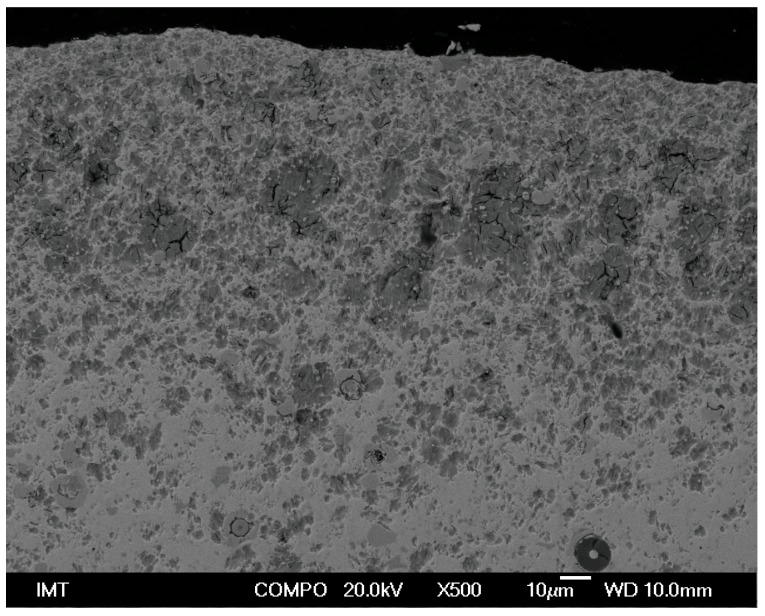
Microstructure of a Gd cylinder in the extrusion transversal direction showing many non-metallic inclusions and cracks (BSE, polished).

Typical backscattered electrons image of the microstructure of the Gd cylinder in the extrusion transversal direction is presented in Figure 5. Since heavy atoms with a high atomic number are stronger scatterers than light ones, this image recorded with back-scattered electrons contains also compositional information. The presence of many inclusions, but also cracks is obvious.

**Figure 6:**
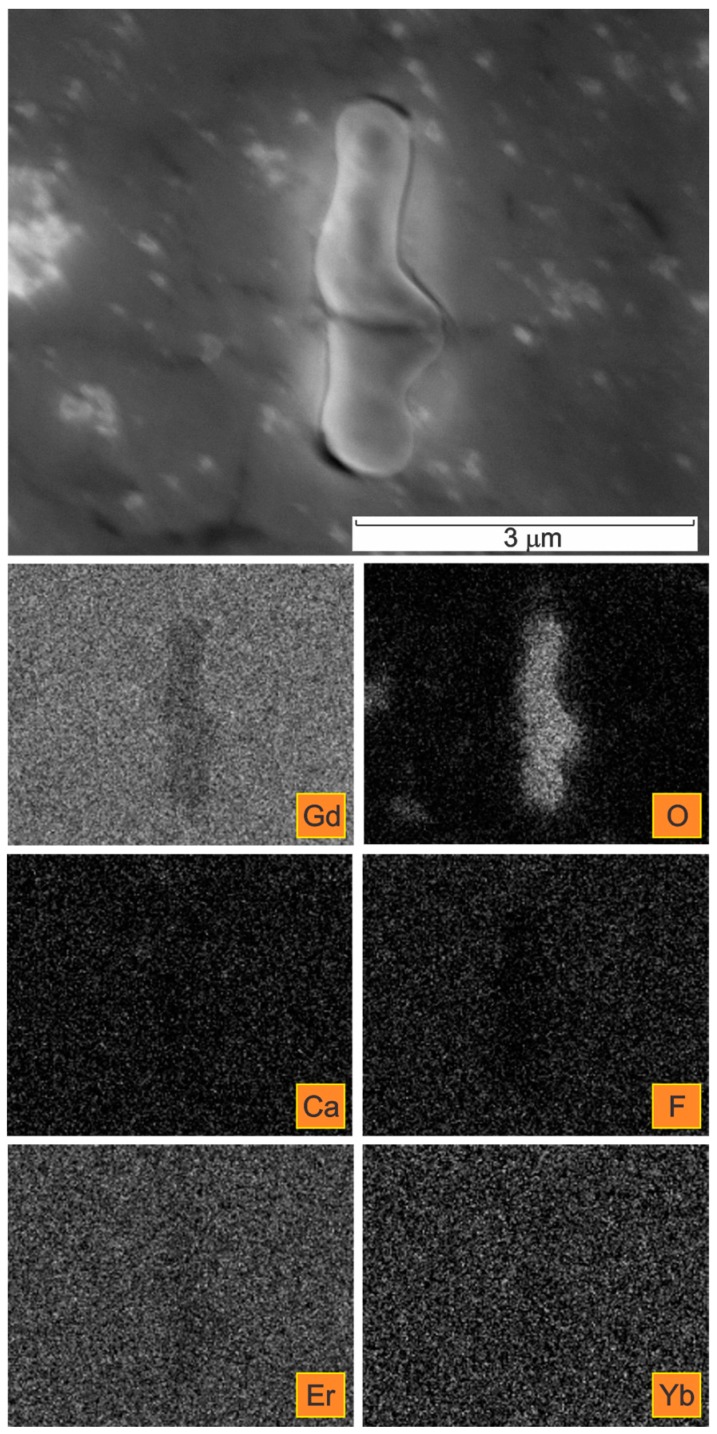
SE image of an oxidized inclusion in a Gd cylinder in the extrusion direction with the corresponding X-ray elemental mappings.

A detailed distribution of the elements in one of the selected elongated inclusions is given in Figure 6. The corresponding X-ray elemental mappings show the distributions of Gd, O, Ca, F, Er, and Yb.

**Figure 7:**
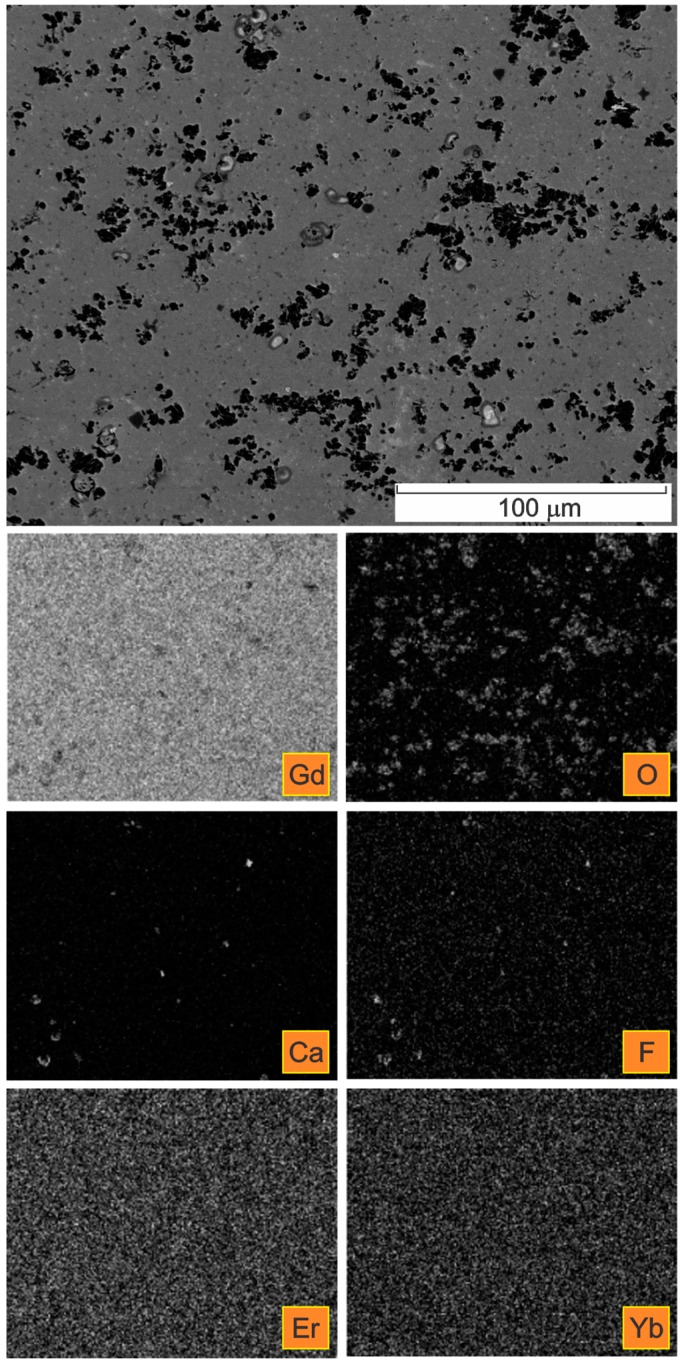
SE image of the microstructure in the centre of a Gd cylinder (extrusion transversal direction) with the corresponding X-ray elemental mappings.

In Figure 7 the microstructure in the centre of a Gd cylinder in the extrusion transversal direction is presented. Complex calcium fluoride inclusions were detected.

**Figure 8:**
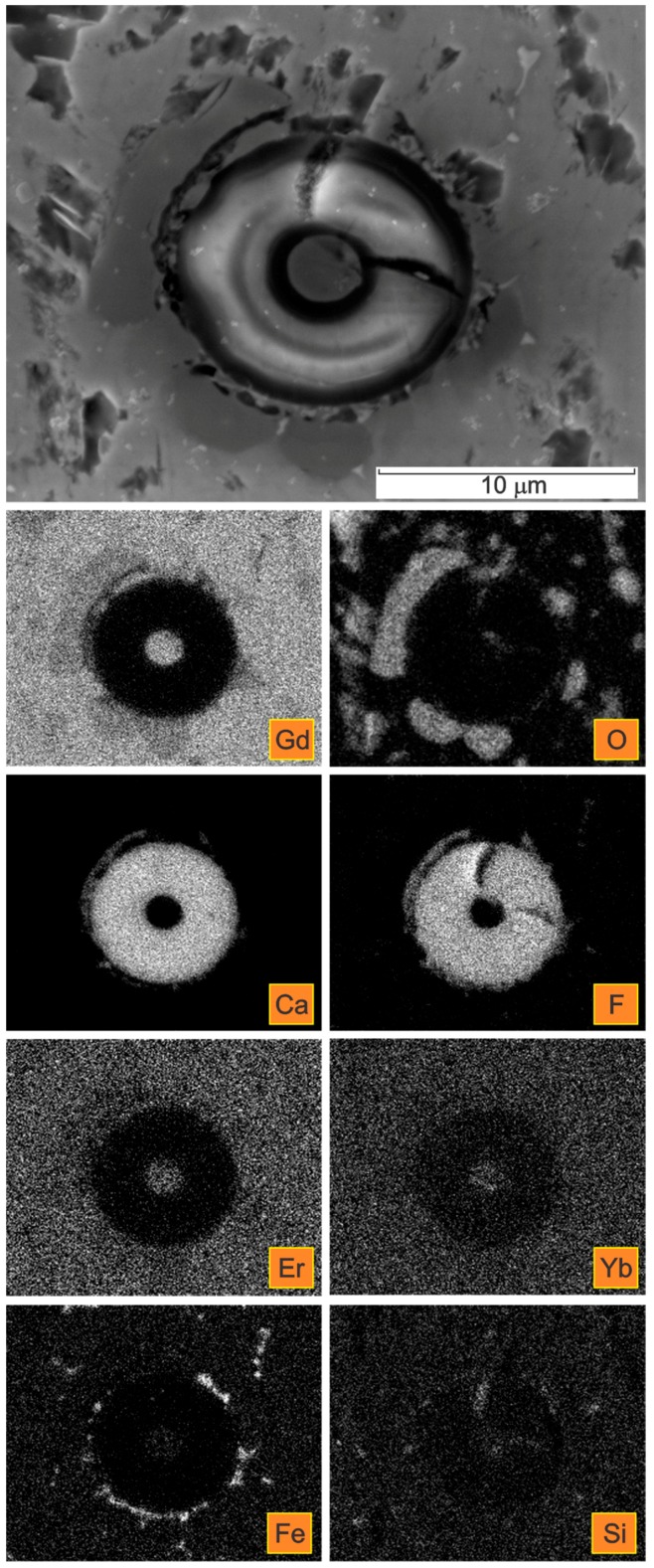
SE image of a CaF inclusion in a Gd cylinder in the extrusion transversal direction with the corresponding X-ray elemental mappings.

**Figure 9:**
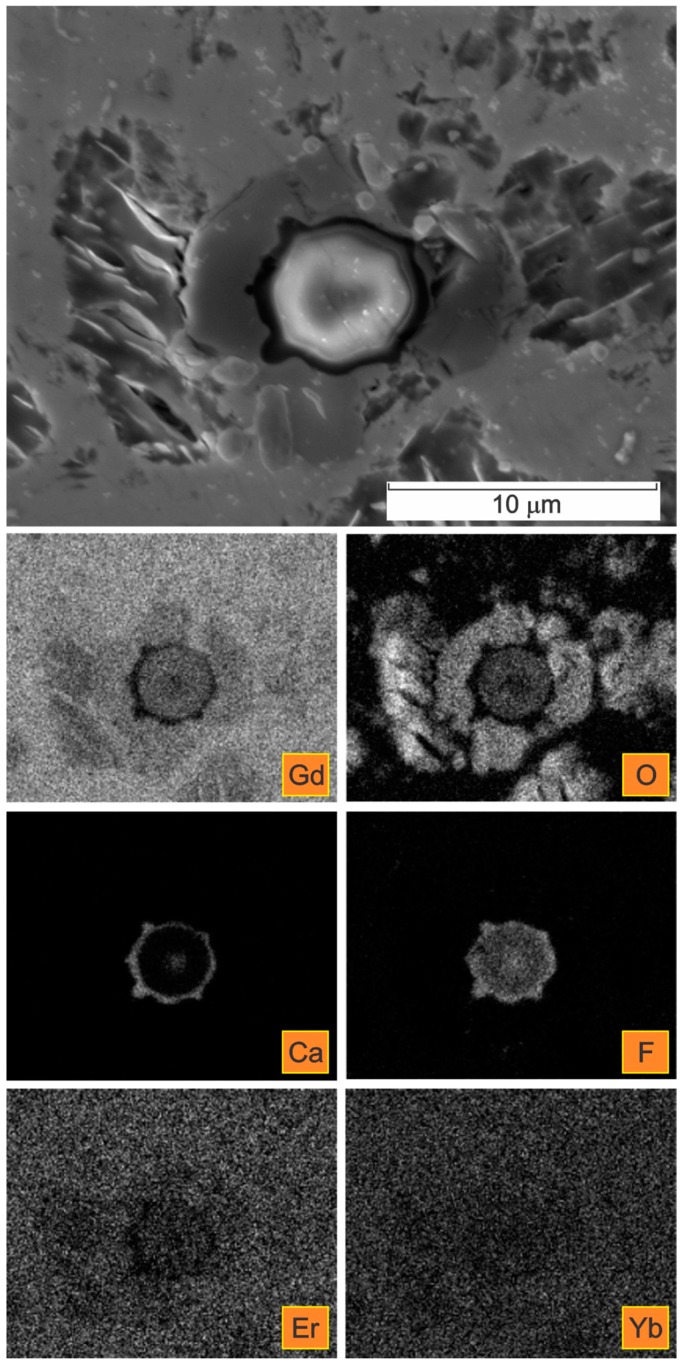
SE image of a CaF inclusion in a Gd cylinder in the extrusion transversal direction with the corresponding X-ray elemental mappings.

The deformable, non-metallic Ca- and F-containing inclusions can be classified as complex calciumoxyfluorides. The oxygen was detected as being co-precipitated with the calcium fluoride inclusions, predominantly forming their outer shell (Figure 6, Figure 8 and Figure 9). The contents of Er and Yb in the calciumfluoride inclusions forming rings are negligible compared to the Gd matrix.

### 3.2. XRD Analysis

**Figure 10:**
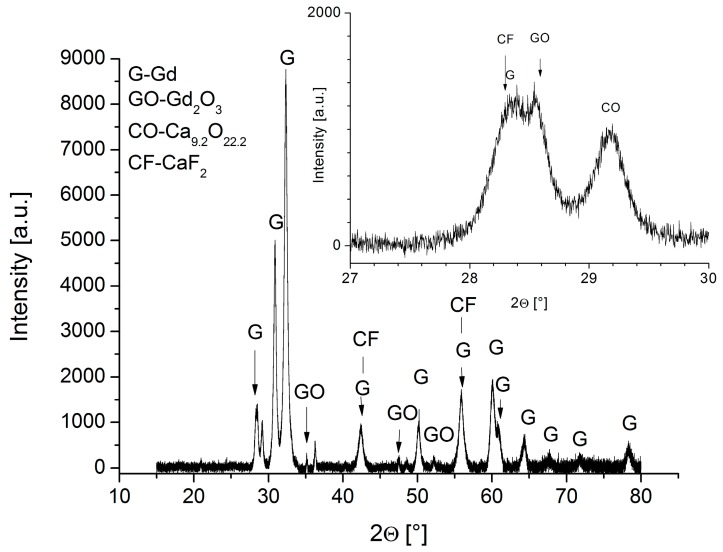
XRD pattern for the extruded Gd-cylinder with insets showing the positions for CaF_2_ and Ca_9.2_O_22.2_.

Acquired XRD spectrum provided us with the information on the phase composition of the Gd-cylinder (Figure 10). Peaks designating gadolinium and Gd_2_O_3_ are clearly visible in the pattern. Extraction of peaks that could be ascribed to CaF, CaO or complex calcium oxyfluorides proved to be very demanding. Nevertheless, we believe that we have been successful in showing the presence of CaF_2_ and Ca_9.2_O_22.2_ which corroborate the results of EDS mapping during the SEM analyses. It has to be noted that the peaks for the both calcium difluoride and calcium oxide bear low peak intensity. This is due to their low atomic number (and, thus, related atomic form factor F) compared to the atomic number in gadolinium and other rare-earth impurities present in our sample. It was estimated from the 2D light microscopy and SEM images that the amount of calcium difluoride and calcium oxide are in the range of 5–10 vol. % yet their footprint in the XRD pattern was very low. Additionally, the peaks for these two phases frequently overlap with those of gadolinium and Gd_2_O_3_ adding complexity to the interpretation of XRD results. Analysis of the peaks showed that these are readily asymmetric which supported the assumption of peak overlapping. Insets in the XRD spectrum should be clear on the positions of the peaks for the CaF_2_ and Ca_9.2_O_22.2_. Peak intensities did not exhibit the expected intensities which could be ascribed to the fact that the Gd-cylinder was initially extruded adding to the effect of texturing of phases within the cylinder.

### 3.3. Mechanical Properties and Flow Curve

**Figure 11:**
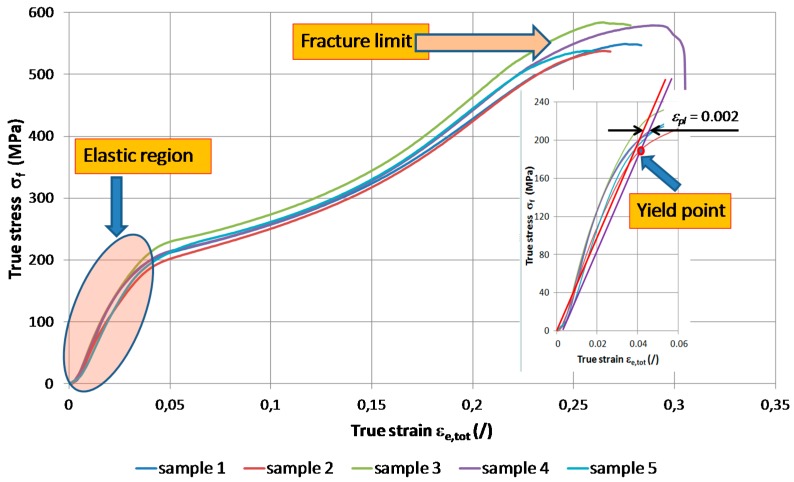
Flow curve of the as-extruded gadolinium and the emphasised elastic part (right).

In order to determine the Young’s modulus of elasticity only the straight part of the flow curve up to the yield point (Figure 11) was analyzed. The calculated Young’s modulus obtained using an evaluation with a linear-regression tool (Equation (3)):

*σ_f_* = *E·ε*_el_ + *k* (3)

showed relatively low values (Table 3) in comparison to the Young’s modulus of gadolinium metal (54.8 GPa) [1]. The inaccuracy between the common equation for the Young’s modulus and the calculated approximation expressed as factor “*k*” is the result of a minor measurement inaccuracy at the start of the experiment. The yield point *σ_f_*_0_ is in the range from 176 to 200 MPa while the maximal true stress before the fracture of the material is in the range from 538 to 583 MPa. The flow curves express slightly concave course up to the strain of 0.23 where they change to the convex shape. Above the strain of 0.267 all samples have cracked along the slip line.

## 4. Discussion

The *ex situ* metallographic examination of the Gd cylinders, which can be used for packed-bed active magnetic refrigerators [8], revealed very inhomogeneous microstructures across the cross-sections of the specimens in both the extrusion and the extrusion-transversal directions. Moreover, a selective oxidation was evident. Especially, surfaces directly exposed to the processing tools during the extrusion and the cutting, are oxidized more intensively.

In addition, the oxygen was found to be co-precipitated with deformable inclusions, predominantly forming their outer shell, as is clear in Figure 6, Figure 8 and Figure 9. 

The elongated Ca- and F-containing inclusions could be classified as complex calcium oxyfluorides. A detailed distribution of the elements Ca, F, and O can be seen in Figure 8 and Figure 9. The detected size of these complex inclusions was up to 6 µm in length and up to 17 µm in diameter.

When used as a magnetocaloric material, high purity Gd is desired [18,19]. Commonly, gadolinium is prepared from gadolinium oxide (99%), and the final metal is obtained using fluorination, reduction, and distillation processes, as well as ultra-purification procedures such as zone refining and solid-state electro transport [18,31].

In the technological routes for metallurgical extraction gadolinia Gd_2_O_3_ is fluorinated with Hydrogen Fluoride (HF) gas in accordance with the following formula (Equation (4)), with GdF_3_ and GdOF as the products:

Gd_2_O_3_(s) + 6HF(g) = 2GdF_3_(s) + 3H_2_O(g) (4)

2GdF_3_(s) + 2H_2_O(g) = 2GdOF(s) + 4HF(g) (5)

Here, Equation (5) is a side reaction [18].

In the reduction phase, the primary product in the above step is reduced by calcium. Among other metallic reducing agents, only calcium forms an oxide that is more stable than that of the rare earths [1]. In the process, the products Gd and CaF_2_ are layered due to the significant density difference, and both of them could be easily separated [18].

The presence of the complex CaF inclusions in the analyzed Gd cylinders confirms the use of the conventional purification processes for gadolinium.

It was further shown that in the complex calcium oxyfluorides inclusions the contents of the rare earths Er and Yb in the CaF phase were negligible, compared to that in the Gd matrix and/or in the oxide phase. This is particularly obvious from the FE-SEM/EDS elemental mappings presented in Figure 8 and Figure 9. This finding is just the opposite of previous reports claiming that the solubility of the rare earths is larger in the fluoride medium than in the oxide [26,32]. A high solubility of rare-earth ions in CaF_2_ has also been reported elsewhere [27].

Regarding the fact that gadolinium metal is a highly reactive element with respect to the air and oxygen, the obtained results of our study are in good agreement with the relevant literature data. Gadolinium forms the monoclinic *β* form of the oxide of the type RE_2_O_3_. As reported by Gupta and Krishnamurthy [1], an increase in the temperature and the humidity accelerates the oxidation of rare-earth metals significantly. The rate of oxidation is considerably increased if the gadolinium contains one or more impurities, such as fluorine, calcium, magnesium, carbon, and iron [1].

In various alloy systems (e.g., steels, aluminum, magnesium, *etc.*) and other inorganic systems (e.g., ceramics, composites) the nature and performance of the inclusions is a relevant topic. A metal matrix with inclusions is microscopically inhomogeneous and can be theoretically considered as a composite. For a better description of the influence of the inclusions on the mechanical properties, the inclusion-matrix interface is of special importance [33].

The obtained results of our study have shown that the deformation processing of Gd enables the selective oxidation of the surfaces and the interfaces (Figure 2, Figure 3, Figure 4, Figure 5, Figure 6, Figure 7, Figure 8 and Figure 9). Moreover, CaF inclusions, as residual impurities after the refining processes, act as active nucleation sites for the heterogeneous precipitation of oxygen. The phenomenon of the internal oxidation of calcium fluorides is evident in Figure 6, Figure 7, Figure 8 and Figure 9.

The results for the Young’s modulus, extracted for the extrusion direction from the elastic part of the stress-strain curve, shows an order-of-magnitude-lower elastic modulus for the gadolinium cylinders than for the pure Gd metal, as quoted in the literature [1,31].

This can be attributed to a lower structural integrity of the material as a consequence of the selective oxidation of surfaces and interfaces of the gadolinium cylinders. Especially when present along grain boundaries, selective oxidation leads to a weakening of the cohesive strength of individual grains [34]. On the other hand, the size range and the quantity of the complex CaF inclusions may also have influenced the integrity of the material, but to a lesser extent.

Due to the complex interplay of different mechanisms, all these factors determine the material’s elastic properties and its formability.

## 5. Conclusions

An *ex situ* metallographic examination of Gd cylinders has shown very inhomogeneous microstructures across the cross-sections in the extrusion and the extrusion-transversal directions. The edges and the outer parts of the cylinders are oxidized more intensively on the surfaces directly exposed to the processing tools during the extrusion and the cutting.

The deformation processing of Gd enables the selective oxidation of the surfaces and interfaces.

A significant morphological anisotropy of the non-metallic inclusions, determined by the direction of the deformation processing, was observed. CaF inclusions act as active nucleation sites for internal oxidation. XRD provided us with the proof that both CaF_2_ and Ca_9.2_O_22.2_ were present in the Gd-cylinder supporting the idea of internal oxidation.

Lower mechanical properties of the material are a consequence of the lower structural integrity due to selective oxidation of surfaces and interfaces of the gadolinium cylinders.

## Figures and Tables

**Table 1:** EDS analyses of the oxidized edges of the Gd cylinder from Figure 3.

Spectrum	Gd	Er	Yb	Si	F	Ca	O	C *
1/wt. %	76.41	0.00	0.00	0.69	0.00	0.00	15.81	7.09
2/wt. %	86.38	4.00	2.99	0.00	0.00	0.00	4.22	2.41
3/wt. %	91.75	0.00	1.87	0.00	0.00	0.00	2.43	2.15

* Total content containing adsorbed C.

**Table 2:** EDS analysis of the Gd cylinder matrix from Figure 4.

Spectrum	Gd	Er	Yb	W	F	Ca	O	C *
1/wt. %	85.00	4.87	2.26	1.17	0.00	0.00	4.03	2.67

* Total content containing adsorbed C.

**Table 3:** Determined E-modulus and yield point in the extrusion direction at room temperature as a result of the analysis of the flow curves.

Parameter	Sample 1	Sample 2	Sample 3	Sample 4	Sample 5	Average
*E* [MPa]	5887	5020	5737	5510	5488	5528
*k* [MPa]	−4.6	−2.2	−1.3	0.3	−10.9	−3.7
*σ_f_*_0_ [MPa]	184	176	200	185	186	186
*R*^2^ [/]	0.9813	0.9924	0.9862	0.9805	0.9915	0.9864
